# Effects of toxic *Microcystis aeruginosa* on the expression of Hox genes in *Daphnia similoides sinensis*

**DOI:** 10.1002/ece3.8685

**Published:** 2022-03-06

**Authors:** Xiaoxue Xu, Yaqin Cao, Huiying Qi, Daogui Deng, Ya‐Nan Zhang, Jianxun Wu, Shuixiu Peng, Zhongze Zhou

**Affiliations:** ^1^ 58286 School of Life Science Huaibei Normal University Huaibei Anhui China; ^2^ 12487 School of Resources and Environmental Engineering Anhui University Hefei Anhui China

**Keywords:** *Daphnia similoides sinensis*, *Hox genes*, maternal effects, *Microcystis aeruginosa*

## Abstract

Lake eutrophication and cyanobacterial blooms have become worldwide environmental issues. Under cyanobacterial blooms (especially *Microcystis*), *Daphnia* spp. can transfer beneficial information to their offspring in order to improve adaptability. *Hox genes* are important regulatory factors of transcription in metazoans, and are involved in the growth and development of organisms. However, the mechanisms of *Microcystis* on the expression of *Hox genes* in *Daphnia* are unclear. In this study, the effects of *Microcystis aeruginosa* on *Hox gene* expression in the mothers and offspring (F1) of two *Daphnia similoides sinensis* clones were investigated using a mixed diet of *M*. *aeruginosa* and *Scenedesmus obliquus*. Compared with the 100%S food treatment, the survival rates at the end of the experiment of clone 1‐F1 in the food treatments containing *M*. *aeruginosa* were significantly lower, but it was significantly higher for clone 2‐F1 in the 20%M + 80%S food treatment. Moreover, the survival rates at the end of the experiment of clone 1‐F1 in the food treatments containing *M*. *aeruginosa* were significantly higher than those of their mother. Based on previous transcriptome data, 14 *Hox genes* of *D*. *similoides sinensis* were identified, including *Abd*‐*B*, *CDX*‐*1*, *Dll*, *HOX*‐*1*, *HOX*‐*2*, *HOXA1*, *HOXA2*, *HOXB3*, *HOXB3*‐*2*, *HOXB7*, *HOXC4*, *HOXC7*, *HOXC8*, and *HOXD10*. The expressions of *Abd*‐*B*, *HOX*‐*2*, *HOXA1*, *HOXC7*, and *HOXD10* of clone 2‐mothers in the 40%M + 60%S food treatment were 2.9–22.5 times as high as in the 100%S food treatment, whereas the expressions of *CDX*‐*1*, *HOX*‐*1*, *HOXB3*, and *HOXD10* of clone 1‐mothers were 4.8–13.1 times at same food level. The expression of *HOXA2*, *HOXC7*, *HOXC8*, and *HOXD10* of clone 1‐F1 in the 40%M + 60%S food treatment was 8.2–21.1 times as high as in the 100%S food treatment. However, compared with the 100%S food treatment, the expressions of *CDX*‐*1* in the mothers and F1 of clone 2 and *HOXB7* in the mothers of clone 1 in the food treatments containing *M*. *aeruginosa* were significantly lower (*p *< .05). Our results suggest that the offspring (F1) produced by *D*. *similoides sinensis* mother pre‐exposed to toxic *M*. *aeruginosa* had stronger adaptability to *M*. *aeruginosa* than their mothers. Moreover, *Hox gene* expressions of *D*. *similoides sinensis* had obvious differences between clones under stress of toxic *M*. *aeruginosa*.

## Graphical abstract

There were significant differences in the survival rates of *D*. *similoides sinensis* under different treatments. Cyanobacteria may be able to affect the expression of *Hox genes* in *Daphnia* via maternal effects. *Hox gene* expressions of *D*. *similoides sinensis* had obvious differences between clones under stress of toxic *M*. *aeruginosa*.

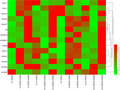

## 1 INTRODUCTION

*Hox genes* are important regulatory factors of transcription in metazoan animals and comprise a large family of highly conserved DNA transcription factors (Affolter et al., 1990). In vertebrates, the *Hox gene* family is often displayed in multiple cluster form, and participates in the regulation of embryonic development and morphological diversity (Krumlauf, 1994; McGinnis & Krumlauf, 1992). In metazoans, the target sites of the *Hox gene* homology domain are connected with specific DNA sequences (Affolter et al., 1990), which can regulate cell fates (Batas, 1993) and affect cell recognition via genetic address (Lawrence, 1992; Lawrence & Morata, 1983). *Hox genes* were first identified in *Drosophila melanogaster* (McGinnis et al., 1984; Scott & Weiner, 1984), and Papillon and Telford (2007) studied the expression and evolution models of *Hox3* and *ftz* genes in *Daphnia pulex*.

Animal mothers can transfer environmental information to their offspring so that their offspring can produce adaptive responses to environmental heterogeneity in terms of phenotype, physiology, behavior, and reproduction (Agrawal et al., 1999; Frost et al., 2010; Mousseau & Fox, 1998). In birds, lizards, insects, and crustaceans, maternal effects play an important role in their population adaptation to the environment (Badyaev et al., 2002; Mousseau & Dingle, 1991; Schwarzenberger & Elert, 2013; Uller, 2004). Boersma et al. (2000) observed that large‐sized *Daphnia magna* could produce larger offspring as well as produce larger ephippia in order to improve their hatching rates. *D*. *magna* can improve net reproduction efficiency and fitness of their offspring after short‐term exposure to the pesticide fenvalerate (Pieters & Liess, 2006). Furthermore, Badyaev (2008) found that the adaptability of a passerine bird to the environment obtained through maternal effects could be preserved for a long time before genetic evolution took place.

In recent decades, cyanobacterial blooms by species such as *M*. *aeruginosa* have become more frequent and severe in lakes due to eutrophication, leading to suppressed population dynamics of various *Daphnia* species (Deng et al., 2008; Hansson et al., 2007; Liess & Hillebrand, 2004; Przytulska et al., 2015). Cyanobacteria often release toxins such as microcystin (MC) which inhibits protein phosphorylation, affects physiological metabolism, and changes chromosomal structure, resulting in genotoxicity (Lankoff et al., 2004; Peng et al., 2018; Zegura et al., 2003). Microcystin (MC) can be accumulated in consumers through the food chain and can even affect human health (Christoffersen, 1996; Gilroy et al., 2000; Jorgensen, 1999; Reynolds, 1994). Usually, *M*. *aeruginosa* has an inhibitory effect on the life‐history traits of *Daphnia* species (Gustafsson & Hansson, 2004; Jiang et al., 2013; Li et al., 2014; Lyu, Meng, et al., 2016; Yang et al., 2011). However, some studies have indicated that single‐cell or small‐colony *Microcystis* spp. can be fed by *Daphnia* spp. to favor their growth and reproduction (Chen & Xie, 2003; Hanazato, 1991; Li et al., 2014). Other studies have even shown that the offspring of *Daphnia* species can obtain more adaptability to toxic *M*. *aeruginosa* via maternal effect (Lyu, Guan, et al., 2016; Lyu et al., 2017). In *Daphnia carinata*, the offspring of the mothers pre‐exposed to *M*. *aeruginosa* had quicker defensive responses than did their mothers previously unexposed to *M*. *aeruginosa* (Jiang et al., 2013). Gustafsson et al. (2005) found that the offspring of *D*. *magna* pre‐exposed to *M*. *aeruginosa* had shorter time to maturation and a greater number of offspring. Schwarzenberger et al. (2009) observed that the offspring produced by the mothers pre‐exposed to *M*. *aeruginosa* up‐regulated the expression of target genes in *D*. *magna*, and suggested that the maternal effect was a short‐term adjustment strategy to the environment.

In summary, *M*. *aeruginosa* could affect life‐history traits and expression levels of some genes in *Daphnia*, but it was unknown how toxic *M*. *aeruginosa* affected the expression levels of *Hox genes* in *Daphnia* species and whether these genes of their offspring from the mother pre‐exposed by *M*. *aeruginosa* had the adaptability to toxic *M*. *aeruginosa*. 14 *Hox genes* have been identified in *D*. *similoides sinensis* based on previous transcriptome data (Zhang et al., 2016), including *Abd*‐*B*, *CDX*‐*1*, *Dll*, *HOX*‐*1*, *HOX*‐*2*, *HOXA1*, *HOXA2*, *HOXB3*, *HOXB3*‐*2*, *HOXB7*, *HOXC4*, *HOXC7*, *HOXC8*, and *HOXD10*. In this paper, our goal is to compare the influences of *M*. *aeruginosa* on *Hox genes* of mothers and F1 in two *D*. *similoides sinensis* clones, and to examine the adaptability of F1 from pre‐exposed mothers to toxic *M*. *aeruginosa* and the differences between two clones.

## 2 MATERIALS AND METHODS

### 2.1 Collection, identification, and culture of *D*. *similoides sinensis*

Lake sediment from the 0‐ to 1‐cm layer was collected from Lake Junshan in Jiangxi province (28°9′41″–28°46′13″N, 116°1′15″–116°33′38″E) in August 2015 using an 8.4‐cm‐diameter columnar gravity corer (Nanjing Institute of Geography and Limnology, Chinese Academy of Sciences). The sediment was washed using 200 mesh (0.074 mm) in the laboratory, and the residue was examined using a microscope (Olympus, Japan) in order to identify the ephippia of *D*. *similoides sinensis* according to the methods of Benzie (2005) and Gu et al. (2013). Ephippia containing resting eggs of *D*. *similoides sinensis* were incubated at 25 ± 1 °C in aerated tap water in an intelligent light incubator (Saifu, Ningbo, China). *S*. *obliquus*, a nontoxic microalgae species, was used as a food source.

### 2.2 Culture of *M*. *aeruginosa* and *S*. *obliquus*

*Microcystis aeruginosa* was obtained from Lake Junshan in August 2015. A single colony of *M*. *aeruginosa* was chosen in the laboratory, and then cultured in BG‐11 medium in an intelligent light shaker incubator (QZB‐98B, China) at (28 ± 1) °C with illumination of a 12:12 h light/dark cycle. *M*. *aeruginosa* which were single or two cells in morphology were collected at the exponential phase of population growth and stored at 4°C.

*Scenedesmus obliquus* was obtained from the Freshwater Algae Culture Collection (Institute of Hydrobiology, Chinese Academy of Sciences), and cultured in BG‐11 medium in an intelligent light incubator (Saifu, Ningbo, China) at 25°C, with a 12:12 h light/dark cycle, then collected at the exponential phase of population growth and stored at 4°C.

### 2.3 *D*. *similoides sinensis* mother experiment

Two *D*. *similoides sinensis* ephippia containing resting eggs were randomly selected, and then hatched in a 50‐ml beaker in an intelligent light incubator at 25°C with a 12:12 light/dark cycle, respectively. The individual hatched from each ephippium containing resting eggs represented one clone, and each clone was respectively cultured through parthenogenesis. Two clones from different resting eggs were employed in the experiment. Third generation youngs (<12 h old) produced by each clone were used as experimental animals in the mother experiment. Three food treatments were designed based on biomass content: 100% *S*. *obliquus* (100% S), serving as a control, 20% *M*. *aeruginosa* + 80% *S*. *obliquus* (20% M + 80% S), and 40% *M*. *aeruginosa* + 60% *S*. *obliquus* (40% M + 60% S). The total biomass of each food treatment was 40 mg/L wet weight. There were three replicates in each food treatment, yielding a total of 18 experimental groups (2 clones × 3 food treatments ×3 replicates). At the beginning of the experiment, 20 young females (<12 h old) at third generation were randomly placed in each 250‐ml beaker. The culture medium was 200 ml aerated tap water (over 48 h). Therefore, 180 youngs were employed for each clone in the mother experiment. The experiments were carried out in an intelligent light shaker incubator (QZB‐98B, China) at (25 ± 1) °C and 12:12 light/dark cycle. All neonates produced by the mothers in each 250‐ml beaker were promptly removed during the experiment. The survival rates of the mothers were calculated daily and lasted at the end of the 14‐day experiment. The culture medium was replaced every two days before *D*. *similoides sinensis* mothers became pregnant, from which point on it was replaced daily. The cultural density (20 young females) of *D*. *similoides sinensis* and temperature (25°C) in this experiment are according to our previous experimental designs (Peng et al., 2018; Xu et al., 2018).

On the fourteenth day, 12‐h‐old neonates produced by the mother in the 20% M + 80% S food treatment were removed and placed in new 250‐ml beakers for an offspring (F1) experiment. At the end of the mother experiment, all *D*. *similoides sinensis* mothers in each food treatment were pooled into an EP tube and stored in liquid nitrogen for later measurement of *Hox genes*.

### 2.4 *D*. *similoides sinensis* F1 experiment

In the mother experiment, owing to fewer offspring produced in the 40% M + 80% S food treatment, the offspring (<12 h old, F1) produced by the mothers of two *D*. *similoides sinensis* clones in only the 20% M + 80% S food treatment on the fourteenth day were collected and regarded as experimental animals in the F1 experiment, and 180 individuals (F1) in each clone were employed. The F1 experimental designs were the same as described in the mother experiment. After 14 days, all F_1_ females in each food treatment were pooled into an EP tube and stored in liquid nitrogen for later measurement of *Hox genes*.

### 2.5 RNA isolation and cDNA synthesis

Total RNA of all mothers and offspring (F_1_) of *D*. *similoides sinensis* in the experiments was extracted using the MiniBEST universal RNA kit (TaKaRa, Dalian, China). DNase I in the kit was used to avoid genomic DNA contamination. A spectrophotometer (NanoDrop™ 2000, Thermo Fisher Scientific, USA) was used to check the concentration and purity of RNA. Total RNA samples were stored at ‒80°C. Single‐stranded cDNA templates were synthesized using the PrimeScript™ RT kit (TaKaRa, Dalian, China), and cDNA template samples were stored at ‒20 °C.

Quantitative real‐time PCR of *D*. *similoides sinensis* *Hox genes* was performed in a LightCycler^®^ 96 PCR device (Roche, Switzerland), using a 2×SYBR^®^ Preix Ex Taq kit (Tli RNase H Plus; TaKaRa, Dalian China). The 10 μL RT PCR reaction contained 5 μL of 2×SYBR^®^Premix Ex Taq (Tli RNaseH Plus), 1.0 μL of the DNA template (1 ng/μL), 0.2 μL of each upstream and downstream primer (10 μM), and 3.6 μL of ddH_2_O. The amplification conditions consisted of an initial step for one cycle of 30 s at 95°C, followed by 40 cycles at 95°C for 5 s and 60°C for 20 s. Fluorescence was measured using a melting curve from 55°C to 95°C in order to detect single gene‐specific peaks and primer‐dimer peaks. The qRT‐PCR primers (Table 1) were designed using Beacon Designer 7.9 (PREMIER Biosoft International, CA, USA), and the results were analyzed using LightCycler^®^ 96 SW 1.1 software. *D*. *similoides sinensis* *Hox gene* expression was quantified using the Q‐Gene method in Visual Basic software based on Microsoft Excel. DsimGAPDH (glyceraldehyde‐3‐phosphate dehydrogenase) and DsimACT (actin) were selected as reference genes (Muller et al., 2002; Simon, 2003). Three biological replicates were used for each sample.

**TABLE 1:** The qRT‐PCR primer sequences of *D*. *similoides sinensis* in the experiment

Name	Sequence	Name	Sequence
*HOX*−*1*‐F	CACGGGTAATTCGCAATC	*HOX*−*1*‐R	GTAGTCGGGTTTGATGTTG
*CDX*−*1*‐F	TTCCATTACAGTCGCTACA	*CDX*−*1*‐R	TTTCTTCACGCTTCTTCAC
*HOXA2*‐F	AATATGGAGAGGTTGCTACT	*HOXA2*‐R	TGACGAATGCTGTTGTTG
*HOXC7*‐F	CATCATCAGCATCATCACAA	*HOXC7*‐R	GCGATGGCTTGATTGTATT
*HOXB7*‐F	GCAACAACAGCAACATCA	*HOXB7*‐R	CAACAGCTACGTCTATGC
*Abd*‐*B*‐F	GCGGATGAAGAACAAGAAG	*Abd*‐*B*‐R	GATGATGATGGTGATGATGG
*HOXB3*‐F	GGCACGGATTCATTCAAG	*HOXB3*‐R	AAGAGGTTGTGATGTTGTTG
*HOX*−*2*‐F	AGAGTACAGTCAGAGTAGTTAC	*HOX*−*2*‐R	CGTTGTGGTGATGATGAG
*Dll*‐F	ATCGTCTAATAAGCGTGTTG	*Dll*‐R	CAGCGTGATGGATACTTG
*HOXC4*‐F	TTCTCACAATCCAGTCATCT	*HOXC4*‐R	TCTCTTCGGTTCCATTCC
*HOXA1*‐F	CAGCACGGAATACAACAG	*HOXA1*‐R	ACTGAATGGTGGTGATGT
*HOXD10*‐F	CGTTATCGGACCAACAAC	*HOXD10*‐R	GTGATGATGCGGATGATG
*HOXB3*‐*2*‐F	CTATCAGTATCACGGTGAAATG	*HOXB3*‐*2*‐R	GAAGAGATTGAGCGGATTG
*HOXC8*‐F	CCTTCGCTTCGTTGTATC	*HOXC8‐R*	GTCACCGTGTTGTTGTTG
*GAPDH*‐F	TCGTCTCCAATGCTTCTT	*GAPDH*‐R	CGGTCCATCAACAGTCTT
*ACT*‐F	CCATCCACCATGAAGATTAAG	*ACT*‐R	CTCGTCGTACTCTTGCTT

### 2.6 Gene identification and sequence analyses

The homologous genes were searched and compared in NCBI (https://www.ncbi.nlm.nih.gov/). Reading frames and functional domains based on the complete sequence information of these homologous genes were predicted using the ORF Finder (https://www.ncbi.nlm.nih.gov/orffinder/) from the NCBI database. Sequence alignment, similarity, and homology analyses were performed using BLASTX (https://blast.ncbi.nlm.nih.gov/Blast.Cgi) and ClustalX. Molecular weight and isoelectric point were predicted using the Compile pI/Mw in ExPASy software (https://web.expasy.org/compute_pi/) (Table 2). Amino acid sequences of *D*. *similoides sinensis* *Hox genes* were predicted using Primer Premier 5. The phylogenetic tree of *D*. *similoides sinensis* *Hox genes* was constructed using neighbor‐joining in MEGA6 software, and a heatmap was constructed using Hemi software (Druga et al., 2016; Tamura et al., 2013; Xu et al., 2018). The sequences of *D*. *similoides sinensis* *Hox genes* had been uploaded in Dryad Digital Repository (https://doi.org/10.5061/dryad.6hdr7sr2n).

**TABLE 2:** Blastx matches for *D*. *similoides sinensis* *Hox genes*

Gene Name	ORF (aa)	Length (bp)	Complete ORF	PI	MW (kDa)	Best Blastx Match					Frame
						Name	Acc. number	Species	*E* value	Identity (%)	
*HOX−1*	89	730	N	10.45	97.7	Homeobox protein Hox‐C4	JAN74469.1	*Daphnia magna*	8.00E−31	100	−1
*CDX−1*	220	1537	N	9.95	25.12	Homeobox protein CDX−1	JAN85504.1	*Daphnia magna*	1.00E−113	98	1
*HOXA2*	852	2961	Y	6.49	92.36	Homeobox protein Hox‐A2	JAN79144.1	*Daphnia magna*	0	89	1
*HOXC7*	142	654	Y	9.84	15.7	Putative Homeobox protein CHOX−7	KOB75552.1	*Operophtera brumata*	3.00E−48	69	−2
*HOXB7*	373	1124	N	9.22	41.04	Homeobox protein Hox‐B7a	KDR08069.1	*Zootermopsis nevadensis*	8.00E−21	80	3
*Abd‐B*	441	1777	N	9.30	45.37	Putative Homeotic abdominal‐B protein	KZS21965.1	*Daphnia magna*	8.00E−134	99	2
*HOXB3*	709	2131	Y	6.1	77.31	Homeobox BarH 2‐like protein	KZS03900.1	*Daphnia magna*	2.00E−38	85	2
*HOX−2*	222	821	N	7.94	24.05	Putative Homeobox protein Hox‐C4, partial	JAN47684.1	*Daphnia magna*	1.00E−39	98	3
*Dll*	347	1250	Y	8.83	38.44	Homeobox protein Hox‐B1, putative	XP_002431233.1	*Pediculus humanus corporis*	5.00E−30	78	3
*HOXC4*	171	518	N	8.55	19.7	Homeobox protein Hox‐C4	JAN74469.1	*Daphnia magna*	1.00E−70	82	3
*HOXA1*	579	3231	Y	8.88	58.99	Homeobox protein Hox‐A1	JAN46366.1	*Daphnia magna*	1.00E−92	99	2
*HOXD10*	366	1533	N	11.76	57.21	Homeobox protein CDX−1	JAN85504.1	*Daphnia magna*	3.00E−53	94	3
*HOXB3‐2*	127	1024	N	10.22	36.85	Putative homeotic HOX3 protein	KZS03900.1	*Daphnia pulex*	8.00E−33	71	2
*HOXC8*	387	1825	N	10.81	68.22	Predicted: homeobox protein MSX−2‐like	XP_019879395.1	*Aethina tumida*	1.00E−41	78	−2

### 2.7 Statistical analyses

All statistical analyses were performed using SPSS 20.0. Two‐way ANOVA was employed to analyze the influences of food treatment, mother‐F1 generation, and their combinations on the survival rates at the end of the experiment and each *Hox gene* expression of each *D*. *similoides sinensis* clone. For each clone, multiple comparisons (Tukey's HSD) were also used to test the differences of the survival rates at the end of the experiment and each *Hox gene* expression of both mothers and F1 among different food treatments, respectively.

## 3 RESULTS

### 3.1 Survival rates of two *D*. *similoides sinensis* clones under different food treatments

**FIGURE 1:**
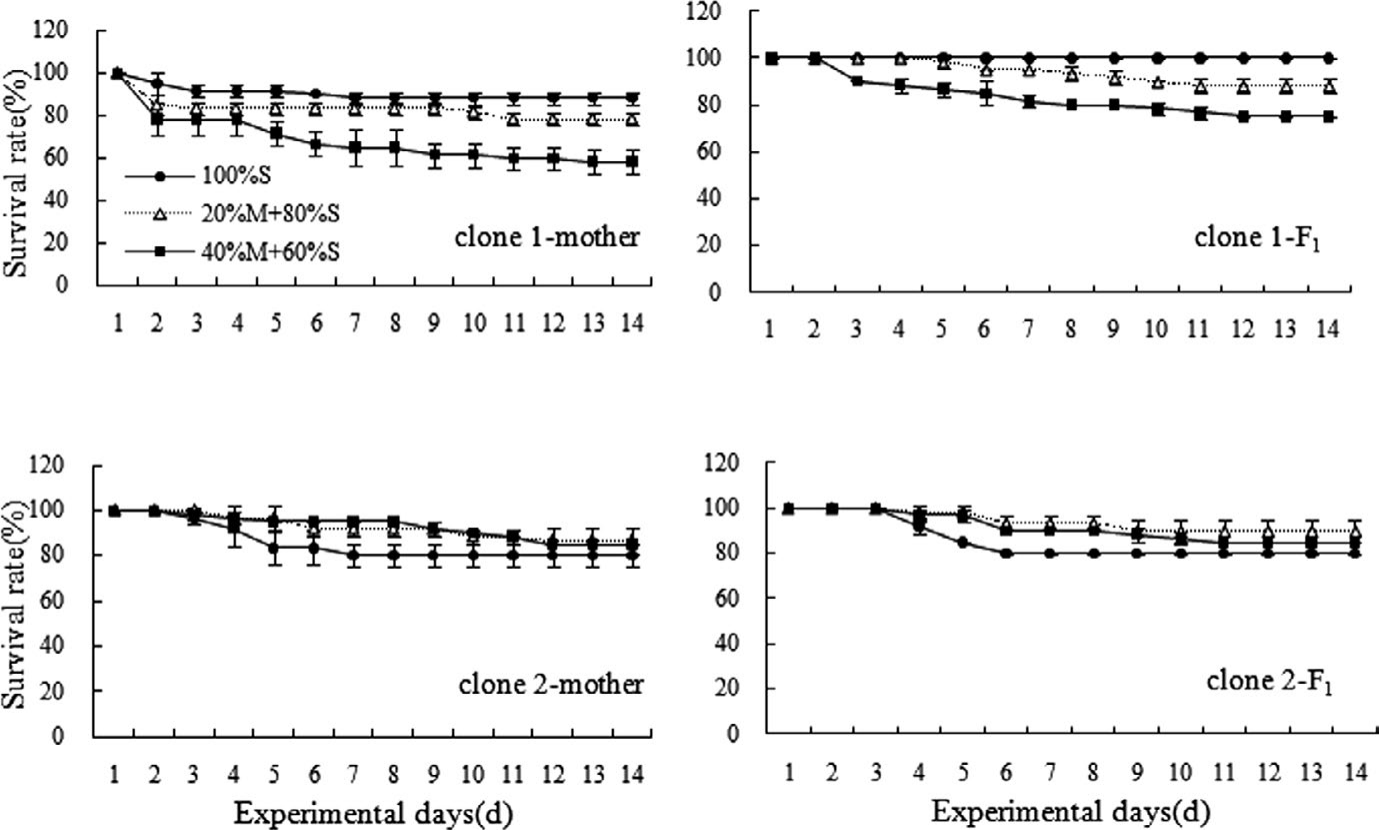
Survival rates of mothers and F1of two *D*. *similoides sinensis* clones under different food combinations of *M*. *aeruginosa* (M) and *S*. *obliquus* (S)

The survival rates of the mothers and F1 in clone 1 showed a gradual dropping trend with the increasing of *M*. *aeruginosa* concentration. However, it was an opposite pattern in clone 2 (Figure 1).

For clone 1, both food treatment and mother‐F1 generation affected significantly the survival rates at the end of the experiment (Food treatment: *F *= 118.429, *p *= .000; Mother‐F1 generation: *F *= 75.571, *p *= .000), but their combinations had no significant effect (*F *= 1.857, *p *= .198). Multiple comparisons (Tukey's HSD) showed that, compared with those in the 100%S food treatment, the survival rates at the end of the experiment of both mothers and F1 in the 40%M + 60%S food treatment were significantly lower (mothers: *p *< .001; F1: *p *< .0001), and it was also significantly lower (*p *< .001) in the 20%M + 80%S food treatment for F1. However, the survival rates at the end of the experiment of F1 in the food treatments containing *M*. *aeruginosa* were significantly higher than those of the mothers (20% M + 80%S: *p* = .0346; 40%M + 60%S: *p* = .0019).

For clone 2, food treatment affected significantly the survival rates at the end of the experiment (*F* = 7.600, *p* = .007), but both mother‐F1 generation and their combinations of food treatment and mother‐F1 generation had no significant effects (mother‐F1 generation: *F* = 0.400, *p* = .539; their combinations: *F* = 0.400, *p* = .679). Multiple comparisons (Tukey's HSD) showed that the survival rates at the end of the experiment of F1 in the 20%M + 80%S food treatment were significantly higher than those in the 100%S food treatment (*p* = .0128).

### 3.2 Identification and characterization of *D*. *similoides sinensis* *Hox genes*

Based on previously published transcriptome data (Zhang et al., 2016), 14 *Hox genes* of *D*. *similoides sinensis* were identified, including *Abd*‐*B*, *CDX*‐*1*, *Dll*, *HOX*‐*1*, *HOX*‐*2*, *HOXA1*, *HOXA2*, *HOXB3*, *HOXB3*‐*2*, *HOXB7*, *HOXC4*, *HOXC7*, *HOXC8* and *HOXD10*, among which *Dll*, *HOXA1*, *HOXA2*, *HOXB3*, and *HOXC7* had complete ORF. The *Hox gene* sequences with the complete ORF‐binding domain covered the entire homeodomain region, and the remainder covered all or part of homeodomain. The 14 sequences consisted of full‐length 89–852 amino acid sequences, with molecular weight (MW) ranging from 17.7 to 97.7 kDa and isoelectric points (pI) ranging from 6.1 to 11.76 (Table 2).

### 3.3 Phylogenetic tree analysis of *D*. *similoides sinensis* *Hox genes*

**FIGURE 2:**
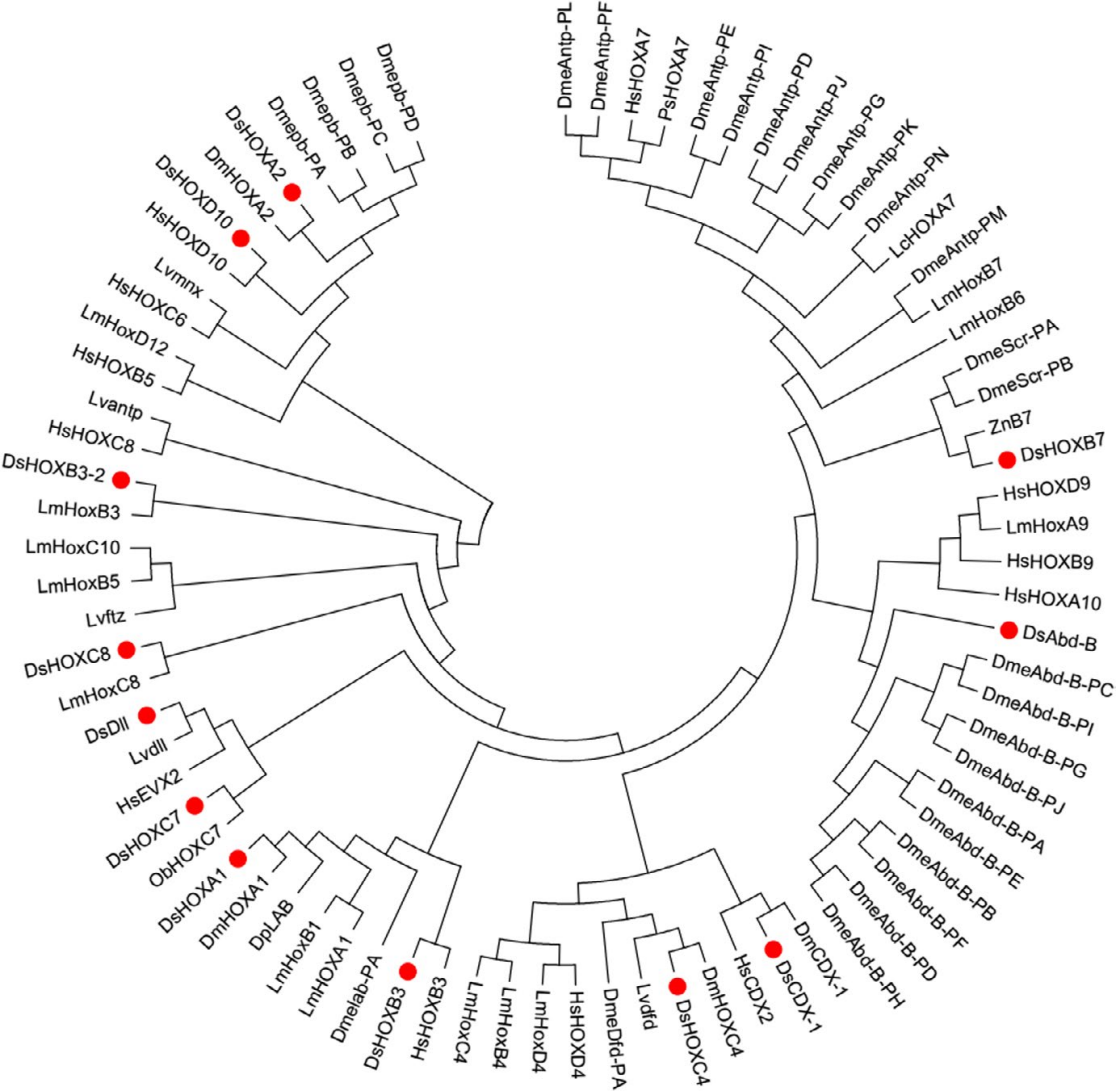
Phylogenetic tree of *Hox genes* in *D*. *similoides sinensis* with other invertebrates and a vertebrate species (Ds: *Daphnia similoides sinensis*, Dp: *Daphnia pulex*, Dm: *Daphnia magna*, Dme: *Drosophila melanogaster*, Hs: *Homo sapiens*, Lv: *Litopenaeus vannamei* (Sun et al., 2015), Lm: *Latimeria menadoensis* (Koh et al., 2003), Ps: *Pelodiscus sinensis*, Zn: *Zootermopsis nevadensis*, Ob: *Operophtera brumate*)

A neighbor‐joining tree of *Hox genes* was constructed based on the amino acid sequences from *D*. *similoides sinensis*, *D*. *pulex*, *D*. *magna*, *Pelodiscus sinensis*, *Zootermopsis nevadensis*, *Operophtera brumata*, *Latimeria menadoensis* (Koh et al., 2003), *Litopenaeus vannamei* (Sun et al., 2015), *Drosophila melanogaster* (http://flybase.org/), and *Homo sapiens* (https://www.genenames.org/). *HOX*‐*1* and *HOX*‐*2* are not included in the phylogenetic tree because of their short amino acid sequences. *Abd*‐*B*, *CDX*‐*1*, *Dll*, *HOXA1*, *HOXA2*, *HOXB3*, *HOXB3*‐*2*, *HOXB7*, *HOXC4*, *HOXC7*, *HOXC8*, and *HOXD10* were respectively clustered into different clades with orthologs in other species (Figure 2).

### 3.4 *Hox gene* expression in the mothers and F1 of two *D*. *similoides sinensis* clones under different food treatments

**FIGURE 3:**
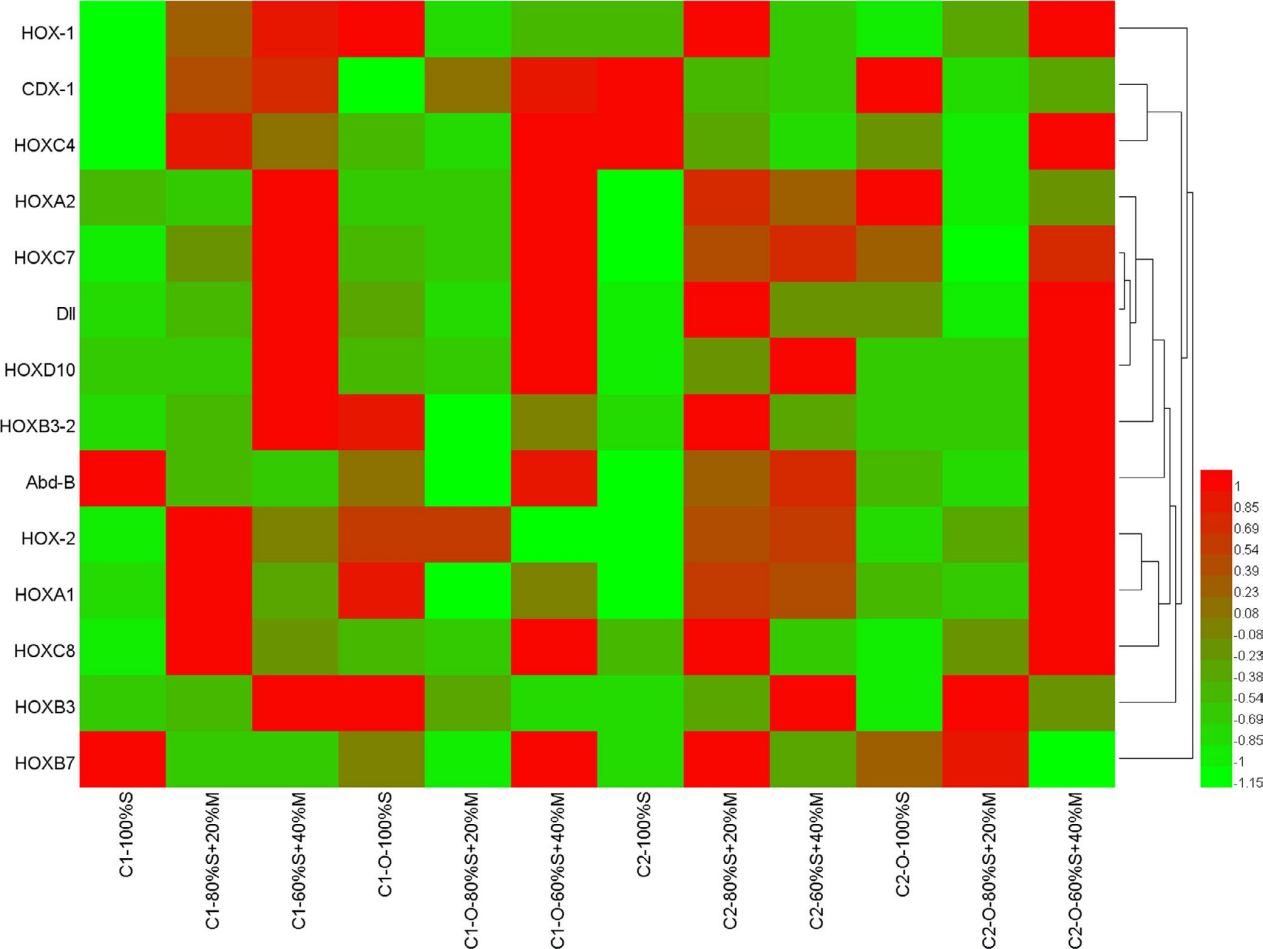
The expression profile of Hox genes of two *D*. *similoides sinensis* clones under three food combinations of *M*. *aeruginosa* (M) and *S*. *obliquus* (S) (C1: clone 1, C2: clone 2, C1‐O: F1 of clone 1, C2‐O: F1 of clone 2)

For clone 1, food treatment and mother‐F1 generation affected significantly the relative expression of *CDX*‐*1*, *HOX*‐*1*, *HOXA2*, *HOXB3*, *HOXB3*‐*2*, *HOXB7*, *HOXC8*, *HOXD10* genes as well as their combinations (Table 3). Moreover, both food treatment and mother‐F1 generation affected significantly the relative expression of *HOXC7* gene (Table 3). In clone 1‐mothers, compared to that in the 100%S food treatment, 11 *Hox genes* (*CDX*‐*1*, *Dll*, *HOX*‐*1*, *HOX*‐ *2*, *HOXA1*, *HOXB3*, *HOXB3*‐*2*, *HOXC4*, *HOXC7*, *HOXC8*, and *HOXD10*) were up‐regulated in the food treatments containing *M*. *aeruginosa* (20%M + 80%S and 40%M + 60%S), whereas the *HOXA2* was only up‐regulated in the 40%M+60%S food treatment (Figure 3). The expressions of *CDX*‐*1*, *HOX*‐*1*, *HOXB3*, and *HOXD10* of clone 1‐mothers in the 40%M+60%S food treatment were 4.8–13.1 times as high as in the 100%S food treatment. Multiple comparisons (Tukey's HSD) showed that the expressions of *CDX*‐*1*, *HOX*‐*1*, *HOXB3*, and *HOXD10* in the 40%M + 60%S food treatment were significantly higher than those in the 100%S food treatment (*p* < .05), whereas the expression of only *HOX*‐*1* in the 20%M + 80%S food treatment was significantly higher than in the 100%S food treatment. Moreover, the expressions of both *HOXB3* and *HOXD10* in the 40%M + 60%S food treatment were significantly higher than those in the 20%M+80%S food treatment (*p* < .05). However, *HOXB7* was significantly lower in the food treatments containing *M*. *aeruginosa* than in the 100% S food treatment (*p* < .05). In clone 1‐F1, the expressions of only *CDX*‐*1* and *HOXA2* were up‐regulated in the 20%M + 80%S food treatment, whereas the other *Hox genes* were down‐regulated. Compared to the 100%S food treatment, the expressions of nine *Hox genes* (*Abd*‐*B*, *CDX*‐*1*, *Dll*, *HOXA2*, *HOXB7*, *HOXC4*, *HOXC7*, *HOXC8*, and *HOXD10*) were up‐regulated in the 40%M+60%S food treatment (Figure 3). The expression of *HOXA2*, *HOXC7*, *HOXC8*, and *HOXD10* of clone 1‐F1 in the 40%M+60%S food treatment was 8.2–21.1 times as high as in the 100%S food treatment. Multiple comparisons (Tukey's HSD) showed that the expressions of *HOXA2*, *HOXC7*, *HOXC8*, and *HOXD10* in the 40%M+60%S food treatment were significantly higher than those in the 100% S food treatment (*p* < .05). In addition, the expressions of *HOXA2*, *HOXB7*, *HOXC7*, *HOXC8*, and *HOXD10* in the 40%M+60%S food treatment were significantly higher than those in the 20%M+80%S food treatment (*p* < .05).

**TABLE 3:** Two‐way AVOVA results on the effects of food treatment, mother‐F1 generation, and their combinations on the relative expression of 14 *D*. *similoides sinensis* *Hox genes*

Clone	Genes	Factors	*df*	*F*	*p*
Clone 1	*Abd‐B*	Generation	1	0.124	.731
		Food treatments	2	1.685	.226
		Generation × Food treatments	2	1.464	.270
	*CDX−1*	Generation	1	44.527	.**000**
		Food treatments	2	6.193	.**014**
		Generation × Food treatments	2	6.153	.**014**
	*Dll*	Generation	1	7.844	.**016**
		Food treatments	2	0.890	.436
		Generation × Food treatments	2	0.042	.959
	*HOX−1*	Generation	1	91.504	.**000**
		Food treatments	2	11.522	.**002**
		Generation × Food treatments	2	12.295	.**001**
	*HOX−2*	Generation	1	2.982	.110
		Food treatments	2	2.701	.108
		Generation × Food treatments	2	2.694	.108
	*HOXA1*	Generation	1	5.555	.**036**
		Food treatments	2	2.730	.105
		Generation × Food treatments	2	3.319	.071
	*HOXA2*	Generation	1	18.463	.**001**
		Food treatments	2	23.563	.**000**
		Generation × Food treatments	2	19.562	.**000**
	*HOXB3*	Generation	1	30.085	.**000**
		Food treatments	2	17.714	.**000**
		Generation × Food treatments	2	18.987	.**000**
	*HOXB3‐2*	Generation	1	12.077	.**005**
		Food treatments	2	4.308	.**039**
		Generation × Food treatments	2	4.694	.**031**
	*HOXB7*	Generation	1	11.046	.**006**
		Food treatments	2	8.550	.**005**
		Generation × Food treatments	2	8.696	.**005**
	*HOXC4*	Generation	1	16.380	.**002**
		Food treatments	2	0.374	.696
		Generation × Food treatments	2	0.421	.666
	*HOXC7*	Generation	1	27.088	.**000**
		Food treatments	2	7.643	.**007**
		Generation × Food treatments	2	0.888	.437
	*HOXC8*	Generation	1	6.456	.**026**
		Food treatments	2	4.080	.**044**
		Generation × Food treatments	2	5.102	.**025**
	*HOXD10*	Generation	1	165.122	.**000**
		Food treatments	2	157.520	.**000**
		Generation × Food treatments	2	97.613	.**000**
Clone 2	*Abd‐B*	Generation	1	5.956	.**031**
		Food treatments	2	4.077	.**045**
		Generation × Food treatments	2	2.508	.123
	*CDX−1*	Generation	1	15.341	.**002**
		Food treatments	2	20.799	.**000**
		Generation × Food treatments	2	1.222	.329
	*Dll*	Generation	1	5.763	.**033**
		Food treatments	2	0.713	.510
		Generation × Food treatments	2	1.337	.299
	*HOX−1*	Generation	1	4.296	.060
		Food treatments	2	1.932	.187
		Generation × Food treatments	2	3.533	.062
	*HOX−2*	Generation	1	5.035	.**044**
		Food treatments	2	1.201	.335
		Generation × Food treatments	2	0.478	.631
	*HOXA1*	Generation	1	8.089	.**015**
		Food treatments	2	4.885	.**028**
		Generation × Food treatments	2	3.994	.**047**
	*HOXA2*	Generation	1	4.765	.050
		Food treatments	2	1.042	.383
		Generation × Food treatments	2	2.221	.151
	*HOXB3*	Generation	1	14.150	.**003**
		Food treatments	2	8.705	.**005**
		Generation × Food treatments	2	7.903	.**006**
	*HOXB3‐2*	Generation	1	7.123	.**020**
		Food treatments	2	5.724	.**018**
		Generation × Food treatments	2	6.038	.**015**
	*HOXB7*	Generation	1	0.099	.758
		Food treatments	2	1.750	.215
		Generation × Food treatments	2	0.578	.576
	*HOXC4*	Generation	1	2.687	.127
		Food treatments	2	0.411	.672
		Generation × Food treatments	2	0.485	.627
	*HOXC7*	Generation	1	11.363	.**006**
		Food treatments	2	2.848	.097
		Generation × Food treatments	2	3.521	.063
	*HOXC8*	Generation	1	15.266	.**002**
		Food treatments	2	16.214	.**000**
		Generation × Food treatments	2	19.089	.**000**
	*HOXD10*	Generation	1	6.262	.**028**
		Food treatments	2	6.530	.**012**
		Generation × Food treatments	2	5.062	.**025**

Note

Bold values indicates *p* <  .05 is significant; *p* < .01 is very significant.

For clone 2, food treatment and mother‐F1 generation affected significantly the relative expressions of *HOXA1*, *HOXB3*, *HOXB3*‐*2*, *HOXC8*, and *HOXD10* genes as well as their combinations (Table 3). Moreover, both food treatment and mother‐F1 generation affected significantly the relative expressions of *Abd*‐*B* and *CDX*‐*1* genes (Table 3). In clone 2‐mothers, the expressions of 10 Hox genes (*Abd*‐*B*, *Dll*, *HOX*‐ *2*, *HOXA1*, *HOXA2*, *HOXB3*, *HOXB3*‐*2*, *HOXB7*, *HOXC7*, and *HOXD10*) in the food treatments containing *M*. *aeruginosa* were up‐regulated compared to that in the 100%S food treatment (Figure 3). The expressions of *Abd*‐*B*, *HOX*‐*2*, *HOXA1*, *HOXC7*, and *HOXD10* of clone 2‐mothers in the 40%M + 60%S food treatment were 2.9–22.5 times as high as in the 100%S food treatment. Multiple comparisons (Tukey's HSD) showed that the gene expressions of *Abd*‐*B*, *HOX*‐*2*, *HOXA1*, *HOXC7*, and *HOXD10* in the 40%M+60%S food treatment were significantly higher than those in the 100%S food treatment (*p* < .05), as were *Abd*‐*B*, *HOX*‐*1*, *HOX*‐*2*, *HOXA1*, *HOXA2*, *HOXC7*, *HOXC8*, and *HOXD10* in the 20%M+80%S food treatment. However, the expression of *CDX1* in the food treatments containing *M*. *aeruginosa* (20%M + 80%S and 40%M + 60%S) was significantly lower than that in the 100%S food treatment. In clone 2‐F1, the expressions of 7 Hox genes (*HOX*‐*1*, *HOX*‐*2*, *HOXB3*, *HOXB3*‐*2*, *HOXB7*, *HOXC8*, and *HOXD10*) in the 20%M + 80%S food treatment were up‐regulated compared to that in the 100%S food treatment. The expressions of 10 Hox genes (*Abd*‐*B*, *HOX*‐*1*, *HOX*‐*2*, *HOXA1*, *HOXB3*, *HOXB3*‐*2*, *HOXC4*, *HOXC7*, *HOXC8*, and *HOXD10*) in the 40%M + 60%S food treatment were up‐regulated compared to those in the 100%S food treatment (Figure 3). Multiple comparisons (Tukey's HSD) showed that the expression of *CDX1* in the food treatments containing *M*. *aeruginosa* (20%M + 80%S and 40%M + 60%S) was significantly lower than that in the 100% S food treatment (*p* < .05), whereas it was only significantly lower in the 20%M + 80%S food treatment for *HOXB3*.

## 4 DISCUSSION

### 4.1 Identification and phylogenies of *D*. *similoides sinensis* Hox genes

In this study, 14 Hox genes of *D*. *similoides sinensis* were identified based on previous transcriptomic data (Zhang et al., 2016; Table 2). In the shrimp *L*. *vannamei*, there were 13 Hox gene protein sequences at the transcriptomic level (Sun et al., 2015). However, 39 Hox gene sequences in *Ichthyophis bannanicus* were found based on genomic data (Wu et al., 2015). Therefore, the 14 Hox genes in *D*. *similoides sinensis* in this study might be underestimated based on the data of the transcriptome rather than the genome.

A phylogenetic tree constructed based on amino acid sequences from vertebrates and invertebrates showed that Hox genes had evolved into different functions after multiple genomic duplication or genomic doubling events. *Abd*‐*B*, *CDX*‐*1*, *Dll*, *HOXA1*, *HOXA2*, *HOXB3*, *HOXB3*‐*2*, *HOXB7*, *HOXC4*, *HOXC7*, *HOXC8*, and *HOXD10* of *D*. *similoides sinensis* were clustered into different clades with orthologs from other species. There was an orthologous relationship between *HOXB3* from *D*. *similoides sinensis* and *HsHOXB3* from *H*. *sapiens* (Sun et al., 2015), and *HOXB3*‐*2* had an orthologous correlation with *LmHOXB3* from *L*. *menadoensis* (Koh et al., 2003). *HOXC4* from both *D*. *similoides sinensis* and *D*. *magna* were clustered into a separate clade with *Dfd* from *L*. *vannamei* (Sun et al., 2015), suggesting that these three species were orthologs. Orthologous relationships between *HOXA1* from both *D*. *similoides sinensis* and *D*. *magna* and *Lab* from *D*. *pulex* were also observed. Moreover, *Abd*‐*B* from *D*. *similoides sinensis* were clustered into a clade with 10 Hox genes from *D*. *melanogaster*.

### 4.2 Effects of food treatment and clone on the survival rate and the Hox gene expressions of *D*. *similoides sinensis*

Usually, the survivals of *Daphnia* are restrained in the presence of *M*. *aeruginosa*. Survival rate and life span of *D*. *galeata* dropped obviously with the increase in *M*. *aeruginosa* concentration (Han et al., 2012). Rohrlack et al. (2001) found that the median survival time of different *Daphnia* species was closely related to their microcystin ingestion rate. In this study, compared with the 100% S food treatment, the survival rates at the end of the experiment of clone 1‐mothers and clone 1‐F1 in the 20%M + 80%S and 40%M + 60%S food treatments were significantly lower, whereas it was significantly higher for clone 2‐F1 in the 20%M + 80%S food treatment. Peng et al. (2018) observed also that the mother exposed to toxic *M*. *aeruginosa* enhanced the fitness of *D*. *similoides sinensis* offspring to *Microcystis* and had the differences among clones. Similarly, different genotypes of *D*. *galeata* showed different tolerance to *M*. *aeruginosa* PCC7806 (Druga et al., 2016). However, Dao et al. (2018) found that the survival rates of *Daphnia lumholtzi* offspring from the mothers pre‐exposed to toxic *Microcystis* evidently dropped in spite of transgenerational adaptability to cyanobacterial toxin. Therefore, *M*. *aeruginosa* affecting *Daphnia* survival rates had the differences between species or clones. Moreover, it had potential limitations using only the survival rate to evaluate the adaptability of *D*. *similoides sinensis* offspring to *M*. *aeruginosa* in this study, and more the life‐history parameters should be employed to study the mechanism.

*Microcystis* can affect related gene expression of *Daphnia* spp. (Druga et al., 2016; Lyu et al., 2015; Schwarzenberger et al., 2009; Schwarzenberger & Elert, 2013; Xu et al., 2018). Schwarzenberger et al. (2009) observed that the presence of dietary microcystins led to the up‐regulation of two genes (glyceraldehyde‐3‐phosphate dehydrogenase and ubiquitin‐conjugating enzyme) which involved in the basic metabolism of *D*. *magna*. Some gene expression of *Daphnia* species to toxic *M*. *aeruginosa* showed the differences between clones (Druga et al., 2016; Xu et al., 2018). In this study, in the 40%M + 60%S food treatment, the survival rates at the end of the experiment of clone 1‐mothers were significantly lower than those of clone 2‐mothers (*p* < .05), and the expression of *Abd*‐*B* in clone 2‐mothers was higher than in clone 1‐mothers. In insects, *Abd*‐*B* is able to regulate the development of the posterior nodules (Hou et al., 2004), affecting the ecdysis and survival. Moreover, in this study, Clone 2‐mother and Clone 2‐F1 had similar survival rates under 20%M+80%S food treatment, whereas their Hox gene expression patterns are different under the same condition. Therefore, the expression patterns of Hox genes may be related to the tolerance of *D*. *similoides sinensis* offspring to *M*. *aeruginosa* and have the differences between clones.

*Daphnia* spp. have an inductive defense mechanism against *M*. *aeruginosa*, which can transfer environmental information and tolerance to *M*. *aeruginosa* to their offspring, and reduce the toxic effects of *M*. *aeruginosa* (Gustafsson et al., 2005; Jiang et al., 2013; Schwarzenberger & Elert, 2013). Compared with the mothers unexposed to *M*. *aeruginosa*, the offspring from mothers exposed to *M*. *aeruginosa* have a shorter time to maturation and produce much more offspring, and so had greater fitness for an adverse environment (Gustafsson et al., 2005). Schwarzenberger and Elert (2013) observed that cyanobacterial protease inhibitors could lead to an increase in protease gene expression of *D*. *magna* offspring. Arginine kinase transcript level of *D*. *magna* offspring whose mothers had been previously exposed to *M*. *aeruginosa* were significantly higher than those of mothers fed with pure *S*. *obliquus* (Lyu et al., 2015). The Hox genes, as a family encoding transcriptional regulator, could regulate the growth and development of crustaceans as well as body formation (Hou et al., 2004). *Dll* is an important gene regulating the growth of arthropods (Hou et al., 2004), and could similarly regulate appendage development in insects (Hughes & Kaufman, 2002). Vachon et al. (1992) found also that the abdomen appendages in insects might not be developed if *Dll* was inhibited by other Hox genes. In this study, compared to those in the 100%S food treatment, the expression of *Dll* of clone 1‐mothers and clone 1‐F1 in the 40%M+ 60%S food treatment was up‐regulated, suggesting that the increasing expression level of *Dll* may protect the development of *Daphnia* appendages. This result may be consistent with which the survival rates at the end of the experiment of clone 1‐F1 was higher than that of their mothers in the 40%M + 60%S food treatment. Moreover, compared to the 100% S food treatment, the gene expression of *Abd*‐*B* and *HOXB7* of clone 1‐F1 were up‐regulated in the 40%M + 60%S food treatment, but down‐regulated in clone 1‐mothers, suggesting that these offspring (F1) may have greater tolerance than their mothers under higher *M*. *aeruginosa* concentration.

## 5 CONCLUSIONS

In this study, 14 Hox genes of *D*. *similoides sinensis* were identified based on previous transcriptome data, including *Abd*‐*B*, *CDX*‐*1*, *Dll*, *HOX*‐*1*, *HOX*‐*2*, *HOXA1*, *HOXA2*, *HOXB3*, *HOXB3*‐*2*, *HOXB7*, *HOXC4*, *HOXC7*, *HOXC8*, and *HOXD10*. In clone 1‐mothers and clone 1‐F1, the survival rates at the end of the experiment of *D*. *similoides sinensis* in the food treatments containing *M*. *aeruginosa* were significantly lower than those in the 100%S food treatment (*p* < .05). Moreover, the survival rates at the end of the experiment of clone 1‐F1 in the food treatments containing *M*. *aeruginosa* were higher than those of the mothers. However, there were no significant differences in the survival rates at the end of the experiment of *D*. *similoides sinensis* clone 2‐mothers between the 100%S food treatment and food treatments containing *M*. *aeruginosa* (*p* > .05). Compared to the 100%S food treatment, the expression of *Abd*‐*B* in clone‐2 mothers was significantly higher in the 40%M + 60%S food treatment, whereas they were down‐regulated in clone 1‐mothers. Therefore, it is likely that the down‐regulation of *Abd*‐*B* in clone 1‐mothers might be responsible for a significant decrease in the survival rates at the end of the experiment under higher *M*. *aeruginosa* concentrations.

The expressions of *Abd*‐*B*, *HOX*‐*2*, *HOXA1*, *HOXC7*, and *HOXD10* in clone 2‐mothers in the 40%M + 60%S food treatment were significantly up‐regulated compared to that in the 100%S food treatment, whereas the expressions of *CDX*‐*1*, *HOX*‐*1*, *HOXB3*, and *HOXD10* were significantly up‐regulated in clone 1‐mothers. Moreover, the expressions of *HOXA2*, *HOXC7*, *HOXC8*, and *HOXD10* of clone 1‐F1 in the 40%M + 60%S food treatment were significantly higher than those in the 100%S food treatment. However, compared with the 100%S food treatment, the expressions of *CDX*‐*1* in clone 2‐mothers and clone 2‐F1 and *HOXB7* in clone1‐ mothers in the food treatmentscontaining *M*. *aeruginosa* were significantly lower. Our results suggest that the offspring (F1) produced by *D*. *similoides sinensis* mothers pre‐exposed to toxic *M*. *aeruginosa* had stronger adaptability to *M*. *aeruginosa* than their mothers. Moreover, Hox gene expressions of *D*. *similoides sinensis* had obvious differences between clones under the stress of toxic *M*. *aeruginosa*. Although our experimental results are satisfactory and rational, it has the potential limitations to reveal the adaptability of *D*. *similoides sinensis* offspring to *M*. *aeruginosa* in the study when we only compared F1 from the mothers in the 20%M + 80%S food treatment with the 100%S food treatment. Therefore, further studies need to be promoted in the future.

## CONFLICT OF INTEREST

All authors declare that they have no conflict of interest.

## AUTHOR CONTRIBUTION

**Xiaoxue Xu:** Conceptualization (equal); Data curation (equal); Formal analysis (lead); Investigation (lead); Methodology (equal); Software (equal); Visualization (equal); Writing – original draft (lead); Writing – review & editing (equal). **Yaqin Cao:** Investigation (supporting); Methodology (supporting). **Huiying Qi:** Investigation (supporting); Methodology (supporting). **Daogui Deng:** Conceptualization (equal); Data curation (equal); Formal analysis (supporting); Funding acquisition (lead); Methodology (equal); Project administration (equal); Supervision (equal); Validation (supporting); Writing – original draft (supporting); Writing – review & editing (equal). **Ya‐nan Zhang:** Conceptualization (equal); Formal analysis (supporting); Methodology (equal); Software (equal); Supervision (equal); Validation (supporting). **Jianxun Wu:** Investigation (supporting); Methodology (supporting). **Shuixiu Peng:** Investigation (supporting); Methodology (supporting); Visualization (supporting). **Zhongze Zhou:** Conceptualization (supporting); Project administration (equal); Supervision (equal).

## Data Availability

Fourteen Hox gene sequences are identified in *D*. *similoides sinensis* through the previous transcriptome data (Zhang et al., 2016. https://doi.org/10.1038/srep34241). Reading frames and functional domains were predicted using the ORF Finder (https://www.ncbi.nlm.nih.gov/orffinder/) from the NCBI database. The sequence data of *D*. *similoides sinensis* in this study have been deposited in Dryad Digital Repository (https://doi.org/10.5061/dryad.6hdr7sr2n).
